# The Immune System as a Biosensor of Health and Athletic Performance

**DOI:** 10.1111/sms.70258

**Published:** 2026-03-17

**Authors:** Stefan Markus Reitzner, Petter Brodin

**Affiliations:** ^1^ Department of Physiology and Pharmacology Karolinska Institutet Stockholm Sweden; ^2^ Department of Women's and Children's Health Karolinska Institutet Stockholm Sweden; ^3^ Department of Immunology and Inflammation Imperial College London London UK; ^4^ Medical Research Council, Laboratory of Medical Sciences Imperial College Hammersmith Campus London UK; ^5^ Pediatric Rheumatology, Astrid Lindgren Children's Hospital Karolinska University Hospital Stockholm Sweden

**Keywords:** elite athletes, energy allocation, exercise immunology, immune health, j‐shaped curve, life history theory, systems immunology

## Abstract

Elite‐level physical performance pushes the boundaries of human physical capacity. The immune system extensively contributes to this effort by its continuous maintenance of skeletal muscle and other active tissues, orchestrating post‐exercise recovery, and as an important integrated sensor network throughout the body. A complexly regulated system with wide ranges of in‐ and outputs, cutting‐edge technologies can enable an increasingly individualized approach without the need for translation of results to human biology. The systems immunology idea of resource allocation plays a major role throughout the human life cycle, and great attention should be directed at the prioritization of these resources to either acute physical performance or immune function, particularly in injury‐ or disease‐prone contexts such as elite athletics. As such, this review aims at emphasizing the systems immunology perspective on immune system function and human performance, and its integration with other systems and factors relevant in physical activity such as nutrition, recovery, and sleep. It also attempts to revisit the previous concept of a J‐shaped curve relationship between performance levels and infection risk. Finally, it will point out key questions that remain unanswered, such as the implications of life‐long training on the composition of the immune system, resource allocation in the competition between immune function and acute exercise, and what implications such adaptations might have for athletic performance. Such an updated, integrated, systems‐level perspective on immunity may guide future research about improved recovery in athletes and more individualized exercise training approaches.

## 1 Introduction

In the pursuit of progressively better performance, elite athletes are pushing the limits of human physiology by improving training techniques, equipment, and physical optimization. Similar to medical science, there is a strong trend toward increasingly personalized interventions to optimize physical performance. Such efforts require the involvement of all organ systems in the body. One important system that has not yet received the attention it deserves is the immune system.

The importance of a balanced immune system in athletic performance has been known for more than a hundred years, a concept that was revisited and put into historical context in the 90s by Nieman 1997 [1], and more recently by Nieman and Wentz in 2019 [2]. One of the first investigations of human immune responses to physical activity was published as early as 1902, investigating leukocyte count changes before and after running the Boston marathon [3]. Since then, only a few studies have been published in the field until the middle of the 1980s, with an opinion by Simon (1984) [4] at the beginning of the modern interest in the field but also casting doubt on the idea of exclusively beneficial effects of exercise. Recently, advancements within the field of immunology have led to a transition from reductionist, animal model‐based research that requires careful interpretation before being applicable to humans, to a more holistic, systems‐level perspective in humans, eliminating the need for the translation of results. However, while the large‐scale deployment of modern omics‐based methods certainly elevated systems immunology to a new level of possibilities, it needs to be pointed out that systems immunology is not simply the application of these technologies to immunological problems, but an entirely different perspective on the functioning of the immune system that focuses on cell–cell interactions and the regulatory networks behind them. This is especially important considering discrepancies between mouse and human immune systems. Some of them are well‐described, including differences in leukocyte subpopulations, Th1/Th2 differentiation or T‐ and B‐cell signaling pathways [5]. With a systems immunology concept in mind that must consider limited resources, trade‐offs and functional prioritization in different periods of life, seasons or even on a circadian basis, the use of animal models becomes even more unfeasible as these aspects fundamentally differ from the human experience.

As a crutch to overcome such species differences, large resources had to be developed to enable translation of animal to human findings [6], highlighting the amount of work required to justify the utility of animal models in immunology. Nevertheless, many findings in exercise immunology do stem from such reductionist approaches, but the importance of advanced, systems‐level human research in the future must be emphasized, also to validate hypotheses arising from animal studies and improve our understanding of species differences.

To investigate how the immune system responds to different kinds of physical activity, an obvious approach is to perform a comprehensive analysis of immune system composition and function with a perturbative intervention, for example before and after an acute bout of exercise. The chronic adaptations that occur in response to exercise, i.e., the physiological differences between high‐level athletes and untrained individuals, are based on a long history of repeated acute exercise bouts that cause a slow shift in baseline state of, e.g., the immune system [7, 8, 9]. Thus, an improved understanding of acute effects can facilitate interpretation of chronic effects.

Human immunity is a complexly regulated system of pattern recognition, cellular dynamics and tolerance with extensive plasticity and production capabilities across the life cycle. It is modulated by a wide range of systemic signals and external inputs, such as pathogens, hormone levels, injuries and physical activity. One way it can be modulated by exercise relates to the allocation of limited energy resources, specific energy substrates and the balance that must be achieved between different functions in the body such as growth, reproduction, maintenance and physical activity, which are differently important in different parts of the life cycle. As such, the immune system is in a constant state of competition for resource allocation with other systems of the body providing these basal functions [10, 11]. From an energetic point of view, the immune system can be a very costly function to maintain [12, 13], particularly in high‐demand situations such as during an ongoing infection or injury, but also during recovery from acute exercise. The involvement of the immune system in the normal physiological recovery from acute exercise is frequently overlooked as one of its core competences. Such “housekeeping” functions could be argued to be equally as important as defense against pathogens. Alternatively, an energy‐depleted system such as following exhausting acute exercise could have less energy resources remaining to spend on immune function, consequently resulting in suboptimal immune responses or impaired recovery from the acute bout of exercise. From the athlete's perspective, two key aspects are fulfilled by an optimized and flexible immune system: 1. reduction of down‐time due to illness, enabling more training sessions during the training season [14] and 2. an improved facilitation of the immediate training adaptation, a process in which structures and systems of the body that are insufficient to meet the performance requirements are broken down and built back stronger. Such flexibility does exist and is required in other aspects, for example during early immune system development, when a tolerance is built for resident digestive system microbiota, or during early life development, where large amounts of energy and resources are directed toward growth [15]. As the conditions of life are constantly changing, its different phases result in different priorities impacting both performance and immunity. However, how these transitions and priorities are regulated over time and transition into each other is still unclear and our understanding of immune system development would be greatly aided by a longitudinal investigation of immune responses across different stages of childhood and adolescence.

The effects of exercise on individual immune components will, over time, result in larger shifts in immune system function. Decrypting such changes appropriately will enable a more individualized approach to health and performance in both athletes and patients, but also in the use of exercise as medicine.

## 2 From Acute to Chronic Adaptations: Accumulation of Molecular Effects Over Time

Acute physical exercise results in a fast re‐balancing of molecular processes to enable active tissues to perform at their physiological maximum. These activated systems set into motion a cascade of complex and interacting signaling events—a “molecular choreography of acute exercise” [16], for example fluctuations in blood pressure, redirection of blood flow, the mobilization of energy substrates and hormone release [16, 17, 18, 19, 20]. Together, these result in leucocytosis and a redistribution of immune cells to the periphery [8]. The exact mechanisms of mobilization can depend on the specific cell type. For example, in 2018 Graff et al. showed that β2‐adrenergic signaling mobilizes, among other NK cells, central and effector memory CD8+ T cells, gamma delta T cells, and non‐classical monocytes. But notably, the mobilization of classical monocytes, naive CD8+ T cells, and CD4+ T cells does not require this catecholamine signal [20]. In the same study, the use of nadolol to blunt exercise‐induced blood pressure fluctuations only partially removed lymphocyte mobilization, leaving at least a partial contribution of a hemodynamic effect. This partial activation can also hint at differential immune system activation based on exercise intensity or type of exercise, resulting in different levels of physiological activation and observable extent of immune system response. Beyond the mobilization of cells, the tight control over this molecular choreography is further visible by a distinct upregulation of pro‐inflammatory cytokines such as most prominently IL‐6, followed by a strong anti‐inflammatory signal with mediators such as IL‐10 and IL‐1RA [21, 22]. A recent meta‐analysis of immunoregulatory myokines in response to acute resistance exercise largely supported these findings based on 34 original studies and additionally for TNF‐a and IL‐15, but conversely not for IL‐10 [23], which leaves room for further investigations with improved and more consistent study design. These time‐dependent gradients of pro‐ and anti‐inflammatory cytokines orchestrate activity and differentiation of effector cells, for example by switching the overall tendency from Th1 to a Th2‐type response [24]. Further systems such as the endocrine and nervous system intensively cross‐talk with immune function during acute exercise, having, for example, an immune‐suppressive effect in the case of stress hormones at higher exercise intensities [25, 26]. In line with the idea of the immune system as a biosensor of the body, exercise‐regulated cytokines such as IL‐1 can trigger hypothalamic activation, affecting the HPA axis and subsequently the release of glucocorticoids, which feed back to regulate immune activity and systemic metabolism [27]. Together, this provides for a sequence of preparation, activation and recovery tailored to the timepoint in relation to the acute form of exercise and its intensity, and this process is generally reasonably well understood and has, among others, extensively been reviewed previously, for example by Cerqueira et al. and Gonçalves et al., both 2019 [28, 29].

Long‐term adaptations in exercise physiology follow a pattern of a gradual baseline shift following sequences of single bouts of acute exercise [7]. Similarly, the accumulated effects of exercise affect the baseline status from two points of view: 1. The upstream signaling cascade is modulated—hormone levels are optimized, blood pressure changes are adjusted after getting used to the type of acute exercise, the enzymatic layout of energy providing processes is streamlined. 2. The immune system itself is affected, by increasing NK cell activity, contributing to the maintenance of thymic mass and balancing out regulatory and effector immune cells [8, 9, 30]. A regular turnover of immune cells by an increased homing to the bone marrow can also contribute to more capable peripheral sets of immune cells and thus improved immunosurveillance which in consequence delays immune‐senescence as outlined in a previous review by Sellami et al. [31]. These advantages of an accumulation of acute effects into long‐term adaptation mentioned in the literature stand against potential downsides: Some studies have mentioned a transient decrease in the function of immune cells, and Gleeson et al. 2011 have famously reported on an increased susceptibility to upper respiratory tract infections [32] (URTIs), but they are mostly not in the range of clinical immunodeficiency. Previous original research has also demonstrated that periods of overly intense training programming—overtraining—can result in impaired neutrophil and monocyte function, reduced NK‐cell cytotoxicity and lower numbers of interferon‐producing T‐cells [33, 34, 35]. Additionally, the systemic load of the totality of cytokine‐mediated overtraining effects such as tissue injury and (metabolic) stress as reviewed before by Smith [36] can add to a reduced functionality of the immune system via interfering with the physiological signaling cascades. To summarize, excessive, poorly managed high‐intensity training regimens can expose an athlete to a transiently compromised immune function, or in other words: overtraining is bad not only for the energy metabolism and the musculoskeletal system but also for the immune system and the same amount of care and attention should be afforded to it.

While our understanding of both acute and chronic exercise can be based on a growing amount of evidence, to date there is no study that attempted to combine both; but with the recent developments of technologies and research approaches, a non‐reductionist approach with comprehensive blood sampling from humans and a subsequent omics‐based *systems immunological* investigation of these topics should not be impossible to execute. The results from such a study would be able to draw the connections between the growing amounts of knowledge of acute and long‐term implementations of the immune system modulation by exercise. This could for example be a combination of ex vivo and cell culture stimulation experiments in subjects with different athletic backgrounds.

## 3 Advantages and Disadvantages of a “Trained” Immune System

Athletes expose themselves to a rigorous training regimen to improve their physical abilities. As discussed above, this can also have a positive long‐term effect on the immune system, for example by decreasing background inflammatory signals [7, 29]. However, just as the risk of injury and over‐exhaustion can be the negative side of the coin in relation to an improvement in physical ability, a stagnated and consistently energy‐deprived immune system could potentially be the negative consequence of such long‐term adaptation from the immuno‐metabolic perspective. While athletes are generally not malnourished, repeated acute bouts of under‐availability of energy metabolites in the time‐critical context of acute high‐level physical activity could manifest as cumulative effects on overall immune performance.

Such a potential disadvantage has been demonstrated for URTIs by Gleeson et al. 2011 [32]. However, since then, Campell and Turner 2018 [37] have critically reviewed new and old evidence and have put this finding in salivary IgA into context, concluding that it can be an unsuitable proxy for overall immune function as too many confounding variables can contribute to IgA variation. Conversely, more recent results by Drummond et al. 2022 [38] showed that acute exercise increases IgA levels in trained individuals, improving mucosal immunity, but also that baseline levels can decrease with chronic heavy training, which could indicate that a potential immunosuppressive effect would be a consequence of overtraining, further outlining the need for highly customized training programming to hit the right level of intensity and avoid overstepping. However, given the more recent development and increased accessibility of comprehensive, systems‐level technologies such as spectral flow and small‐volume proteomics that can relay a more central representation of immune function, peripheral proxies such as the above‐mentioned IgA might be altogether abandoned, at least in the pursuit of answering fundamental mechanistic questions.

Certainly, the physiological performance improvements of athletes can add to the enhancement of immune function. As an example, by adjusting the enzymatic layout and their mitochondrial content, high‐level endurance athletes have a highly optimized energy metabolism, enabling their physical performance [39, 40], but also potentially providing the large amounts of energy required during homeostasis, mainly fueled by oxidative phosphorylation and during infection or injury events, where the additional energy needed is provided by further glucose‐dependent pathways [41]. Endurance athletes in particular exhibit large adaptations in their ability to metabolize lipids with a high throughput [42], which would greatly aid the maintenance of the immune system. The related field of immunometabolism has gained attention in recent years and its advances have been comprehensively reviewed by Padilha et al. in 2022 [9], where the authors also propose the “vacant space” theory, a mechanism by which senescent (T‐)cells are mobilized to the periphery during acute exercise where they become apoptotic, resulting in a drag of new, younger and less pro‐inflammatory cells and thus a lowered baseline inflammatory status over time [43]. High‐level athletes of specific sports also have largely enhanced muscle mass [44]. While the density of tissue‐resident immune cells such as macrophages does not change in a large dimension [45, 46, 47], the absolute numbers of these cells per muscle would be increased. As more cells are confronted with an antigen, together with increased vascularization in the same individuals [48, 49], this increased number of reporters but also better access to central organs could result in an improved immunization after intramuscular injection of vaccines.

Conversely, during COVID‐19 we observed less severe symptoms in individuals that spent less resources and energy on immune function such as children and adolescents, which did not develop a “cytokine storm” [50, 51]. Extending this logic in the other direction, individuals with very high energy producing capabilities such as high‐level athletes should have been more severely affected, while epidemiological data show the opposite [52]. This might indicate a tighter regulatory control against over‐ and under‐shooting the immune response.

Even with the use of modern technologies allowing for the measurement of more direct measurements such as spectral flow or mass cytometry, it has to be kept in mind that the transient reduction in circulating peripheral immune cells such as NK cells, neutrophils, and macrophages in response to acute exercise [53, 54] might have different, more physiological reasons. Acute exercise is known to cause plasma dilution during recovery [55, 56], which would relatively decrease the concentration of all immune cells. Despite being high‐resolution and throughput methods, investigating circulating immune cell populations and cytokines will furthermore always be a snapshot of what is largely a transition tissue—blood—and not the target tissue such as skeletal muscle or lung mucosa. A reduction in observed cell numbers could thus be a representation of the increased infiltration of target tissues by immune cells. Another possible mechanism could be an increased reactivity of specific cells, such as higher degranulation of NK cells in young athletes, as shown previously [57]. Accordingly, a true overview of acute exercise immune effects would require a study design sampling not only blood but also target tissues and potentially central organ tissues. While central organ biopsies might be a challenge in humans, skeletal muscle biopsies are frequently sampled in exercise physiological research, and future investigations would benefit not only from a multi‐omics but also from a multi‐tissue approach to acute exercise immune effects.

## 4 The Immune System Integrated Into Physiological Homeostasis

The performance of an elite athlete is a product of the optimization of not only the immune system but also other key active and supportive tissues and systems in the body. Body composition is improved, energy production capacity increased, the endocrine system is optimized, and the nervous system, regularly pushed to its limits, is highly adapted. These systems enable and support each other in their function in a cross‐talk manner. As such, a lack in one system's function can restrain and limit overall performance.

One key factor in high‐performance athlete programming is nutrition, which sometimes has to conform to weight class conditions, or needs adjustment in sports where body weight plays a role. A lack of key nutrients such as iron, zinc, or vitamins such as A, D, E, B6, or B12 can substantially increase the risk of infection and dysfunction as previously reviewed by Gleeson 2016 [58]. Another review by Gleeson [53] highlighted the importance of a sufficient glucose supply to maintain immune cell function, but that an excess of energy substrates such as saturated fats can have a suppressive effect. As an example, a high‐fat diet based on saturated fats was recently found to impair IL22 secretion by a subset of intestinal innate lymphoid cells leading to dysbiosis and increased gut permeability, interestingly, an effect that was not observed in a high fat diet based on unsaturated fats [59]. Gut health can thus affect the efficiency with which nutrients can be taken up. This example highlights how energy substrates are not equally available in all, which can have substantial consequences on athlete health. Further preliminary results from our own lab also show that as a long‐term consequence, subcutaneous white adipose tissue (scWAT) gene expression in high‐level endurance and strength athletes shows reduced lipid‐associated macrophage signatures, indicating a reduced level of baseline inflammation in scWAT (*unpublished data*).

Such specific modulatory effects are not exclusive to fats, but there also exists evidence that not all carbohydrates have the same effect on the immune system, and that timing matters. Especially during acute exercise, high availability of carbohydrates can reduce the exercise‐induced increase in cortisol levels and pro‐inflammatory cytokines, with Chen et al. 2008 showing that this effect is most pronounced when ingesting pre‐exercise carbohydrates in sufficient quantity [60]. A recent study outlined this great importance of carbohydrates as a source of energy for the immune system by demonstrating that a “low carb high fat” diet of comparable caloric value does not carry the same benefits [61]. This was also recently reviewed in a carbohydrate‐focused review by Bao et al. [62], however, not with elite athletes, which showed further indications that the type of carbohydrate—glucose, sucrose or fructose—has a differential influence on specific pro‐ and anti‐inflammatory cytokines, despite the overall carbohydrate quantity being the main factor.

Another factor is protein ingestion, and the dose recommendations have been regularly changing and depend on activity level. For immune system function, high levels of dietary protein intake of 3 g/kg body weight have been shown to be superior to an energy‐matched diet with 1.5 g/kg body weight protein content in preventing respiratory infections [63]. A recent randomized controlled trial had similar results, with post‐workout meals consisting either of protein‐carbohydrate or carbohydrate only over a 10‐week training period, with the protein‐carbohydrate group showing a lower post‐exercise pro‐inflammatory status [64].

While as discussed above the strictly caloric value of the ingested food only is not sufficient, a lack of energy will negatively affect immune system function. However, how the body prioritizes energy expenditure and potentially reserves to perform at least the basic immune system functions remains to be investigated. There are indications that immune cells adapt to chronic endurance exercise by increasing their mitochondrial content, supporting the notion of their energy metabolic sensitivity, and suggesting a high‐level athlete immune cell phenotype [65, 66, 67]. Together, this paints a picture of a substantial influence of nutrition on the function of immune cells that have to be factored in for high‐level athletes. Special attention has to be given to athletes in sports with specific weight classes, or where bodyweight plays a role, but also when special diets have to be observed. Low bodyfat proportions in female athletes, for example, not only cause menstrual dysfunction but can also have a decisive negative influence on energy availability during training and competition settings [68]. The choice of nutrients but also other environmental factors will further have a large impact on the composition of the microbiome, which in turn is essential in the synthesis of B vitamins in the gut. The proper integration of the gut microbiome at this important interface of the “outside” and the “inside” of the body is also a highly interesting question of early life development, which might play a role in laying a solid early foundation for enabling exceptional physical performance later in life. For example, tryptophan metabolites from microbiota can modulate T‐cell differentiation in newborn children, creating a direct link to the immune system [69]. Disruption of the microbiota can impair both microbial production and host absorption state, which can lead to deficiency states despite sufficient intake of dietary precursor metabolites and vitamins [70, 71].

A non‐optimized nutrition can have further consequences in other physiological aspects, for example hormone balance in the endocrine system. Testosterone and estrogen are both hormones based on the chemical structure of cholesterol and would therefore be negatively impacted in their biosynthesis by insufficient ingestion of fats—again in the right balance between unsaturated and saturated. This can have large consequences for the pro‐inflammatory or autoimmune tendency of the immune system, as recent results from our own lab showed [72]. In brief, the immune systems of individuals that underwent a gender‐affirming hormone treatment with testosterone became more pro‐inflammatory compared with before the treatment but also showed less autoimmune tendencies. Energy availability will also affect the levels of stress caused during acute exercise and as a baseline background via the HPA‐axis, consequently increasing—or decreasing—cortisol levels. Certain levels of cortisol increase the availability of glucose, modulates inflammation and facilitates cardiovascular exercise‐readiness [73]. In high‐performance athletes, that can over time also result in higher stress resilience due to a blunting of the inducibility of the HPA‐axis by non‐exercise stressors [74]. However, on the other hand, an over‐activation of the HPA‐axis, for example with overtraining or with insufficient recovery can result in impaired performance and increase injury risk [75, 76]. The high relevance of stress of all kinds on physical performance, as well as on immune system function, is further underlined in the 2019 IOC consensus statement about mental health in athltes [77].

An important factor in recovery from stress is sleep, which has a beneficial effect on the HPA axis and consequently on the immune system. This has previously been reviewed by van Dalfsen and Markus 2018 and Irwin 2019, pointing out that sleep supports, among others, homeostasis in cytokine signaling, and disturbance will result in dysregulation [78, 79]. Chronic sleep loss will result in increased ACTH and cortisol baseline levels and subsequently increased stress reactivity [80]. This negative effect continues into the immune system with an increase in pro‐inflammatory cytokines such as IL‐6, resulting in higher injury risk and impaired muscle recovery [81, 82].

## 5 Bench to “Track‐Side”‐a Translational and Future Perspective

The immune system provides a unique window into the body, an integrated system that is present in all parts of the body [83]. If properly interpreted, it can be used for early detection of physiological shortcomings or the readiness for physical performance levels. Better understanding of the underlying processes behind immune system perturbation, basically a translation matrix for observable features of the immune system, could further be developed into a clinically useful biosensor, already uniquely deployed to every corner of the body, to monitor health and physiological adaptation in athletes. As such, improving our understanding of how the immune system works in the context of physical activity might have a synergistic effect on medical and health‐related applications beyond athletes. As a side note, physical activity has previously been shown to alleviate symptomatics in several immune‐system related diseases such as rheumatoid arthritis, lupus erythematosus or multiple sclerosis [84, 85, 86]. A current comprehensive review by Simpson et al. highlighted that moderate, regular exercise enhances immune defense [87]. However, to reach such a deeper level of understanding, future research is required to focus on the interactions between the immune and other systems of the body under *physiological* conditions. A distinction could be made by investigating differences in the immune response to acute exercise in sedentary subjects compared with high‐level athletes, in essence the *physically active* human as a “model organism” for the non‐perturbated human immune system. However, it has to be noted that athletes often participate in supra‐physiological training, resulting in overreaching and overtraining, pathological‐adjacent processes that can make it harder to isolate purely physiological effects. Further granularity could be provided by taking different exercise backgrounds of these athletes into account. A typical adaptation of endurance athletes lies in their increased energy metabolic capacity [88, 89], while strength‐based athletes which typically perform short bouts of exercise improve mainly in their contractile force and the tissue involved in this aspect [90, 91], both with different consequences on the starting conditions of the immune system. Unveiling these factors layer by layer will gradually increase the granularity of the interpretation of this personalized biosensor. To this, a layer of disease‐related mechanisms could be added, such as vaccinations or pathogen confrontation. As an example, we could consider an “acute” version of life history theory—a limited amount of available (energy‐)resources can be spent at any given time. As discussed above, acute exercise mobilizes the immune system, thus the readiness to confront pathogens or antigens, but simultaneously also directs energy resources away from immune functions. This perspective is one of systems immunology rather than of a disconnected mechanistic investigation of individual aspects of the immune system in model organisms. A future research question could be where on this spectrum the ideal balance of physical activity lies for an optimized immune or immunization response, a question that will be hard to answer for human biology in different species, or with an isolated view on individual mechanisms without considering complex cell–cell interactions and their regulatory networks. This would certainly also be tied to timing—what phase of an infection, or how long before or after a vaccination would have what degree of effect. More recently, some evidence for the benefit of light‐to‐moderate acute exercise before vaccination in enhancing antibody responses emerged, an effect that seems to be enhanced with age [92, 93], while there is also evidence for an increased risk for adverse reactions of post‐vaccination acute exercise [94]. However, further research, especially into the interaction between acute and long‐term physical activity and pathogen‐mediated infectious diseases is required to understand the regulatory background of such a resource allocation. While this would only take energy metabolic factors into account, both physical activity and immune function can be limited not only by calories available but also the lack of individual nutrients, trace elements, vitamins. How would limitations in any of these important factors be managed when prioritizing either physical performance or immune function? These questions certainly make the case for an increased collaboration between classical immunology and exercise physiology, providing an exceptional opportunity to observe the immune system in action under *physiological*, not pathological conditions. Such future research will form the basis for biomarkers that can be used in the above‐mentioned biosensor‐approach.

### 5.1 The J‐Shaped Curve Reviewed and Revisited

Although there is still much to be unveiled regarding biomarkers and resource management in the life history theory and systems immunology perspective, accumulated new evidence and understanding does warrant an update of other classic concepts such as the controversial J‐shaped curve of exercise‐induced immune suppression. The first half of this concept—the beneficial effect of moderate‐intensity exercise over no exercise on the immune system is well established [8, 37, 95]. Counteracting this positive influence is the potentially negative influence of over‐reaching or over‐training [34, 35], which becomes increasingly relevant with increasing exercise load. During the conception of this idea, some findings on epidemiological and mechanistic levels initially supported the idea of an increasing infection risk with increasing levels of activity. For example, decreasing circulating lymphocyte numbers with acute exercise [96], reduced NK‐cell activity [97], or increased incidence of infections among athletes [98].

However, these observed effects can be explained at least in part by mechanisms such as a post‐exercise plasma dilution effect [55, 56], or confounding factors such as the increased contact of athletes with people traveling and competing at international competitions as mentioned in the recent IOC consensus statement [99]. On a mechanistic level, there is also newer evidence that actually shows increased functionality of NK cells due to higher degranulation tendency [57].

With this increasing amount of additional evidence, an update of the original J‐shaped curve concept based on extrapolating the increasing exercise effect and projecting an increasingly negative effect on the immune system, which was already controversial at its conception, was warranted. In their adequately entitled publication “Debunking the Myth of Exercise‐Induced Immune Suppression”, Campell and Turner concluded that the post‐exercise decrease in lymphocyte numbers and function rather reflects a transient re‐distribution of immune cells to peripheral tissues, which *increases* immune surveillance rather than decreasing it [37], a concept already supported by Lancaster and Febbraio in 2015 [100]. An S‐shaped curve has been proposed where athletes at the elite end of the spectrum show a lower susceptibility to infections by simply re‐evaluating previously published data [101].

This *epidemiological* indication that the J‐shaped curve requires an update can also be resolved with a more systemic perspective view on immunology rather than focusing on individual mechanisms. The original concept of a J‐shaped curve fails to appreciate that higher levels of performance can only be achieved with optimization of the physiological systems reviewed above, that enhance both immune function and physical performance. Certainly, higher exercise loads can increase the risk of infection and injury and involve potentially serious risks such as life‐threatening rhabdomyolysis, as well as the risk for small, over time accumulating issues can be increased [102]. However, elite‐level performance routinely requires optimization of auxiliary physiological systems to be reached. Amateur‐level athletes might run the risk of occasionally stepping into an increased immunological risk by pushing performance boundaries without the accompanying optimization required, elite athletes can run the risk of performing on their usual, high level but lowering their control over other systems' physiological optimizations. These excursions carry increased risk, but the consequences are self‐regulating and hopefully self‐adjusting in their influence on training programming.

### 5.2 A Systems Perspective on Exercise Immunology

**FIGURE 1:**
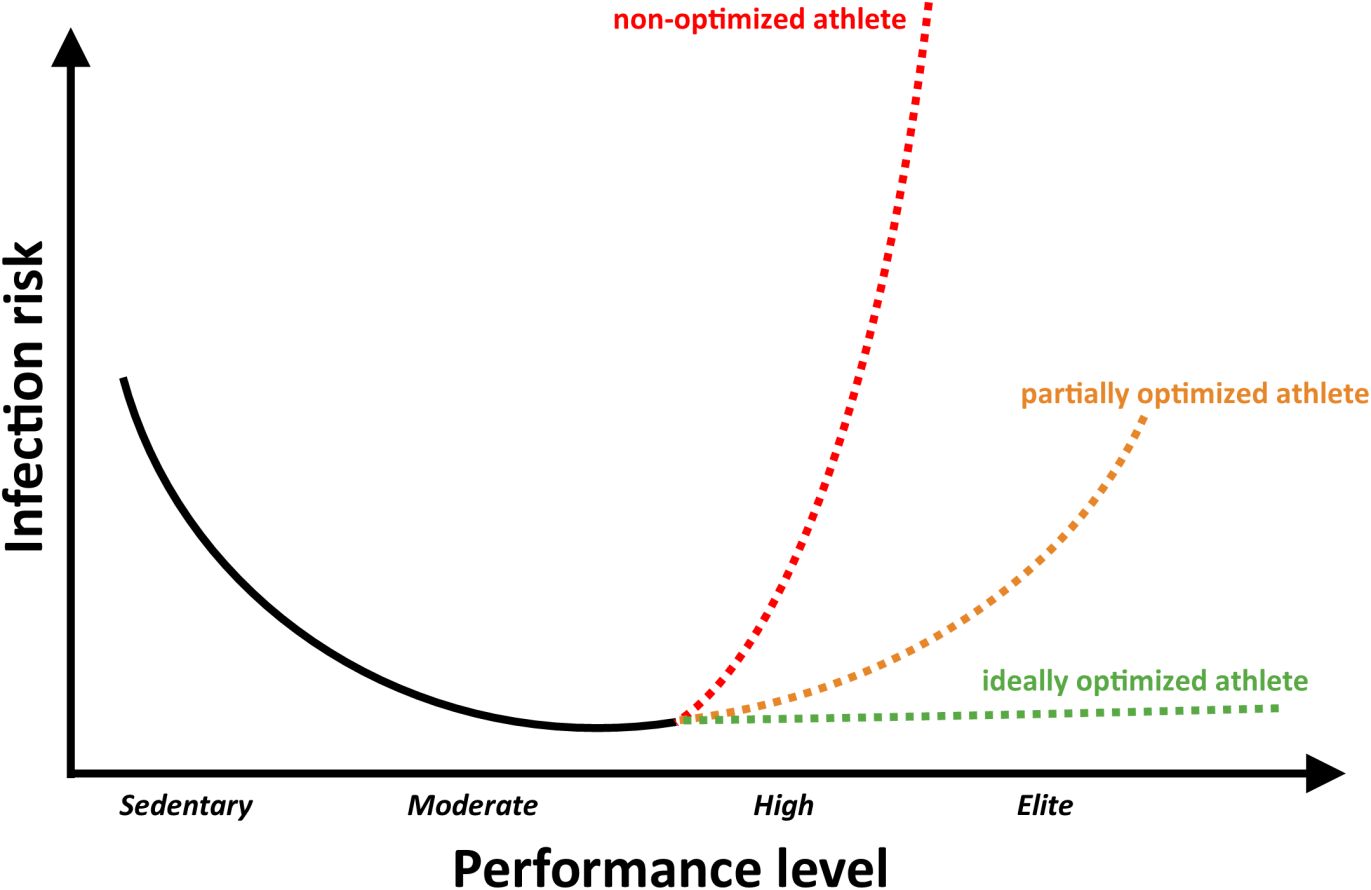
An updated concept of the relationship between performance levels and infection risk. Evidence supporting the upwards trajectory of the original J‐shaped curve concept has been debated perviously [37, 87]. Performance is limited by optimization of the “biological machine athlete” and will not be able to be increased. To reach higher performance levels, optimization needs to take place, which simultaneously has an effect on disease risk.

This modified curve‐concept (Figure 1), the integrated idea of optimized systems supporting each other's function is certainly translatable to amateur athletes. Appropriate, complete nutrition with quality ingredients, sufficient good quality sleep and a reduction of psychological stress will consequently improve immune system function in this population. As the immune system often works with good performance margins and to some extent system redundancies, the need for optimization becomes more apparent with increasing athletic performance. Considering life‐history theory, as introduced above, the improvements that pay off the most might be changing across the life cycle, and as general performance levels decrease with age, the limits for optimization will also be consistently lowering.

We can however derive several adjustments from the literature that can improve immune function. Indications exist that a mix of both endurance and resistance training can be beneficial for immune function [103, 104], of course, within the constraints of the programming required by elite athletes which might limit the maximally achievable benefit. Further, evidence shows that both the quality and quantity of food play a major role, with the important factors being to cover caloric requirements (or at least only a small caloric deficit), to provide appropriate quality, with for example saturated fats having a different effect than unsaturated fats, and to cover essential micronutrients such as iron and zinc [105, 106], and vitamins [107, 108]. Additionally, as during sleep the immune system goes through important regulatory adjustments and can spend more energy on producing cytokines and differentiate immune cells [109], sleep deprivation must be avoided as much as possible. This can be especially difficult for elite athletes who often travel internationally and are challenged by changing sleep environments and time zone effects. To summarize, elite athletes do optimize key systems that improve immune system function, but this improvement is used to “buy” high‐level physical performance. The bottom line of this equation strongly depends on the quality and precision not only of the training programming but also other adjacent adaptations that should be under close monitoring of competent coaches and medical professionals.

This concept can certainly be applied to amateur athletes and individuals that only participate in everyday activities. Just as the tail end of the updated J‐shaped curve can be optimized, allowing for higher performance levels, the left side of the curve can also be adjusted with improved nutrition, sleep and recovery. Amateur athletes can be encouraged to optimize these parameters *before* hitting a performance wall, thus avoiding the risk of over‐training or over‐reaching in the first place. Across the life cycle, the collected evidence so far suggests that the human body is made to move, increasing in its importance with advancing age [110, 111], keeping immune cells functionally younger and more capable, enhancing the immune response to pathogens, injuries and vaccinations. But consequently, as this concept of a trade‐off between high‐level—extreme—performance and health takes shape involving the optimization of the adjacent systems of the body, even ideally optimized athletes will naturally aim for performance at the border of human abilities, regardless of the level of optimization. As such, a key for elite athletes being health and staying healthy is a constant and close monitoring of their health status with all tools available and precise and determined intervention in case parameters of health show an increased risk.

## Funding

The authors have nothing to report.

## Conflicts of Interest

The authors declare no conflicts of interest.

## Data Availability

Data sharing not applicable to this article as no datasets were generated or analysed during the current study.
